# Functional Analysis of Genes Encoding Juvenile Hormone Receptor *Met* and Transcription Factor *Kr-h1* in the Reproductive Capacity of *Coccinella septempunctata* Males

**DOI:** 10.3390/insects16010049

**Published:** 2025-01-06

**Authors:** Ying Cheng, Yuhang Zhou, Cao Li

**Affiliations:** 1 Guizhou Institute of Plant Protection, Guiyang 550006, China; zyh2008484118@163.com; 2 Guizhou Provincial Pollution-Free Engineering Center of Plant Protection, Guiyang 550006, China; lc2019gzdx@163.com

**Keywords:** *Coccinella septempunctata*, juvenile hormone, *methoprene-tolerant*, *krüppel homolog 1*, RNAi, reproductive regulation

## Abstract

This study focuses on the regulatory effects of genes encoding the juvenile hormone (JH) receptor methoprene-tolerant (*Met*) and transcription factor krüppel homolog 1 (*Kr-h1*) on the reproductive capacity of *Coccinella septempunctata* male adults. *Met* and *Kr-h1* expression levels were analyzed in males fed on artificial diets with and without JH by quantitative real-time PCR, and the effects of *Met* and *Kr-h1* on male reproduction were analyzed by RNA interference technology. *Met* transcription levels in 5- and 10-day-old males fed with a JH-supplemented diet were lower than those without JH. *Kr-h1* expression in 5-day-old adult males was lower in diets lacking JH but was higher in 10-day-old males fed on a diet lacking JH. There were no significant differences in the testes sizes of male ladybugs injected with *Met*-dsRNA when compared to *GFP*-dsRNA; however, the testis volume of ladybugs injected with *Kr-h1*-dsRNA was smaller than those injected with *GFP*-dsRNA. After males were injected with *Met*-dsRNA and *Kr-h1*-dsRNA, the mean egg production by females decreased by 12.75% and 23.10%, respectively, at 20 d postinjection. Our results show that *Met* and *Kr-h1* have important roles in regulating reproduction by directly affecting testes development in males and egg production in females.

## Simple Summary

The ladybug, *Coccinella septempunctata* L. (Coleoptera: Coccinellidae), is an important natural enemy of aphids, whiteflies, and jassids. The reproductive capacity of *C. septempunctata* decreases when supplied with artificial diets, which restricts large-scale breeding. Adding juvenile hormone (JH) to artificial diets can significantly increase egg production and hatching rates in *C. septempunctata*. JH is a gonadotropin that is produced and secreted by the corpora allata and has roles in metamorphosis, growth, and reproduction. RNA interference (RNAi) has shown that genes encoding the JH receptor *Met* and transcription factor *Kr-h1* play important roles in female reproduction; however, the impact of *Met* and *Kr-h1* on testes development and reproductive capacity in male ladybugs remains unclear. In this study, we studied the effects of various diets on *Met* and *Kr-h1* expression in male ladybugs, and RNAi was used to verify the regulatory roles of *Met* and *Kr-h1* in the reproductive ability of male ladybugs. Our results further illustrate how JH modulates insect reproduction, which is relevant when considering dietary guidelines for the artificial propagation of ladybugs.

## 1. Introduction

Insect juvenile hormone (JH) is a gonadotropin that is produced and secreted by the corpora allata and has roles in metamorphosis, growth, and reproduction [1,2,3,4]. The genes encoding the methoprene-tolerant (*Met*) receptor and transcription factor krüppel homolog 1 (*Kr-h1*) play important roles in regulating insect reproduction via JH [5,6,7,8]. Knockdown experiments using RNA interference (RNAi) technology demonstrated that the suppression of *Met* or *Kr-h1* expression in stinkbugs, moths, and ladybugs inhibited transcription of the vitellogenin (*Vg*) and vitellogenin receptor (*VgR*) genes and led to reduced oocyte maturation and oogenesis [9,10,11,12]. Lu [13] reported that feeding *Harmonia axyridis* on diets supplemented with JH analogs enhanced the reproductive ability of adults by accelerating ovary development and increasing mating frequency and egg production. Han et al. [14] showed that JH promoted ovary development and upregulated *Met*, *Kr-h1*, *Vg*, and *VgR* expression in *H. axyridis*. The addition of the JH analog methoprene to the *Bactrocera tryoni* diet promoted the growth of ejaculatory ducts and the development of male testes [15]. Furthermore, male insects with reduced levels of JH led to decreased egg production in mating females and reduced numbers of progeny. It is well established that JH stimulates the development of male accessory glands and regulates their secretions, which impacts reproductive behavior [16,17]. JH is also known to regulate the expression of reproductive genes in male insects. For example, when newly emerged male adults of *Agrotis ipsilon* were injected with exogenous JH, the expression of *Met* and *Kr-h1* was induced, which was accompanied by an increased length of male accessory glands; furthermore, knockdown of *Met* and *Kr-h1* expression was associated with a reduction in the lengths of male accessory glands [18]. These results indicate that exogenous JH and JH analogs have functions similar to endogenous JH and target *Met* and *Kr-h1* in the JH signaling pathway to regulate insect reproductive behavior. The large-scale breeding of natural enemies is a popular method of biological control; however, their use is hampered by the high costs of breeding and rearing. Consequently, the development of effective artificial diets for natural enemies is critical [19]. The addition of JH to artificial diets or the increase in JH levels via exogenous treatments can significantly improve reproductive ability, which is of paramount importance in large-scale breeding efforts for natural enemies.

The large-scale, monoculture mode of planting crops and the excessive use of pesticides have led to decreases in the types and populations of natural enemies in the field. Theoretically, the large-scale breeding of natural enemies and the release of supplemental populations will potentially prevent and control insect pests. *Coccinella septempunctata* L. (Coleoptera: Coccinellidae), also known as the ladybug, is a critical natural enemy of leafhoppers, whiteflies, and aphids. Although insect diets are often utilized for rearing ladybugs, this approach is expensive and time-consuming. Artificial diets can decrease the costs associated with rearing and improve uniformity in the ladybug population [20]; however, artificial diets can disrupt the activity of neurosecretory cells and the corpora allata [21]. This can result in lower hormone titers and reduced Vg levels, which ultimately impact ovary development and oocyte maturation and may result in females entering premature reproductive diapause [22].

We previously reported that the addition of JH to the ladybug artificial diet resulted in increased egg production and hatching rates [22]. Furthermore, RNAi technology was used to demonstrate that *Met* and *Kr-h1* play important roles in the reproductive ability of *C. septempunctata* females by modulating Vg synthesis, ovary development, and fertility [20,23]. Furthermore, JH is an important reproductive gonadotropin in insects. Studies on the reproductive function of the JH receptor *Met* and the signaling pathway *Kr-h1* genes are scarce, and only a few studies exist on the regulation of male reproduction in ladybugs. It is important to mention that the impact of *Met* and *Kr-h1* on testes development and the reproductive capacity of male ladybugs remains unclear. In this study, we studied the effects of various diets on the male ladybug expression of *Met* and *Kr-h1*. Furthermore, RNAi was used to verify the regulatory roles of *Met* and *Kr-h1* in the reproductive ability of male ladybugs. Our results further illustrate how JH modulates insect reproduction, which is relevant when considering dietary guidelines for the artificial propagation of ladybugs.

## 2. Materials and Methods

### 2.1. Insects

The ladybugs used in this study were originally collected in wheat fields and were reared indoors on aphids for at least 25, generations as described in [20,24]. Experiments were executed in growth chambers maintained at 70 ± 5% relative humidity (RH) and 25 ± 1 °C with a 16/8 h light/dark photoperiod.

### 2.2. Artificial and Aphid Diets

All ingredients in the artificial diets were sourced locally, and the diets were compounded as described in [22]. The methods for rearing ladybugs on *Aphis craccivora* Koch (Hemiptera: Aphididae) have been reported previously [24].

The components of diet 1 were documented in [22] and included the following: milk powder, 15 g; pig liver, 105 g; eggs, 10 g; olive oil, 2 g; corn oil, 2 g; casein, 7.5 g; cholesterol, 5 g; sucrose, 45 g; protein powder, 4.5 g; powdered yeast, 0.5 g; vitamin C, 1 g; honey, 7.5 g; vitamin E, 1 g; sterile water, 370 g; and agar, 6.17 g.

The components of diet 2 included all substances listed for diet 1 plus 3 μL of 65% juvenile hormone III (JH III, Shanghai ACMEC Biochemical Technology Co., Shanghai, China).

The aphid diets, *A. craccivora*, were maintained on horsebean seedlings in the laboratory [24].

### 2.3. Expression of Met and Kr-h1

Five- and ten-day-old male adults (*n* = 4 for each age) were collected and fed on an aphid diet or diet 1 or 2. Samples were replicated three times for the two ages of male adults, flash-frozen in liquid nitrogen, and kept at −80 °C until needed. The Eastep Super Total RNA Isolation Kit (Promega, Beijing, China) was used to isolate RNA, and cDNA was obtained as previously described [23]. The mRNA sequences of *Met* and *Kr-h1* were previously deposited in the National Center for Biotechnology Information (NCBI) as accessions OR135688.1 and OR183710.1, respectively; these sequences were used to design primers Met-F/Met-R and Kr-h1-F/Kr-h1-R (Table 1). Primers for the ladybug *Actin* gene were designed and synthesized according to Liu et al. [25]. Gene expression in different developmental stages was analyzed using the Met-F/Met-R, Kr-h1-F/Kr-h1-R, and Actin-F/Actin-R primers (Table 1). Quantitative real-time PCR (qPCR) was undertaken in a 20 μL volume that included the following: forward and reverse primers, 2 μL each; cDNA template, 2 μL; Sso Advanced Universal SYBR Green Supermix (Bio-RAD, San Diego, USA), 10 μL; and ddH_2_O, 4 μL. The qPCR reaction conditions included pre-denaturation for 2 min at 95 °C, denaturation for 5 s at 95 °C, and 30 s of annealing and extension at 60 °C for 39 cycles. The melting curve conditions were 65–95 °C in 0.5 °C increments at 2–5 s/step. Relative expression was analyzed using the 2^−∆∆Ct^ method [26].

### 2.4. RNAi of Met and Kr-h1

#### 2.4.1. dsRNA Synthesis

Primers specific for the gene encoding green fluorescent protein (*GFP*) are also listed in Table 1. cDNA from *C. septempunctata* males was used as a template, and DNA fragments specific for *Met*, *Kr-h1*, and *GFP* were amplified as recommended in the Phanta^®^ Max Super-Fidelity DNA Polymerase kit (Vazyme, Nanjing, China). PCR products representing *Met*, *Kr-h1*, and *GFP* were isolated by agarose gel electrophoresis, and DNA fragments were purified using the FastPure^®^ Gel DNA Extraction Mini Kit (Vazyme). The TranscriptAid T7 High-Yield Transcription Kit (Thermo Fisher Scientific, Lenexa, USA) was utilized for reverse transcription. Met, Kr-h1, and GFP double-stranded RNA (dsRNA) were synthesized in a total volume of 40 μL and contained the following: template DNA, 6 μL; 5X Transcript Aid reaction buffer, 8 μL; dNTP mix, 16 μL; Transcript Aid Enzyme Mix, 4 μL; and DEPC-treated water, 6 μL. Reverse transcription was executed at 37 °C for 4 h and then treated with DNase I for 15 min at 37 °C; the reaction was terminated by adding 0.5 M EDTA (2 μL, pH 8.0) for 10 min at 65 °C. Reactions were stored at −80 °C until needed.

#### 2.4.2. dsRNA Injection

Male adults (1-day-old) were microinjected with 1 μL of a stock solution containing 4500 ng/μL of Met-dsRNA, Kr-h1-dsRNA, or GFP-dsRNA. The injection point in male adults was at the internodal region between the 3rd and 4th abdominal segments. Microinjection needles were inserted for 5 s and then removed. Controls consisted of a non-injected group and GFP-dsRNA-injected adult males. Treatments consisted of 50 males, and experiments were repeated three times.

#### 2.4.3. Effects of RNAi on Male Adults

Adult male ladybugs that were microinjected with the three dsRNAs were fed with the aphid diet. On the 4th and 8th d following the extraction of RNA, synthesis of cDNA and qPCR were conducted with male adults on the fourth and eighth day after microinjection with dsRNAs, as described in Section 2.3. Treatments consisted of three samples, and each sample contained four males.

On the 5th and 10th d following microinjection, males were inspected with a stereo microscope equipped with Image View software (x64,4.11.18709.20210403), which was utilized to calculate testes widths and lengths. Thirty dissected males were used in each treatment for imaging.

In another experiment, 10 microinjected males were paired with females emerging on the same day, and the numbers of eggs were counted daily for 20 d. In this experiment, each treatment was replicated three times for a total of 30 mating pairs; controls consisted of males that did not undergo microinjection with dsRNA.

### 2.5. Statistical Analysis

One-way analysis of variance (ANOVA) was used to analyze data, and the multiple comparison least significant difference method was utilized to assess significance with DPS v. 19.05 [27].

## 3. Results

### 3.1. Effects of JH on Met and Kr-h1 Transcription in Adult Male Ladybugs

**Figure 1:**
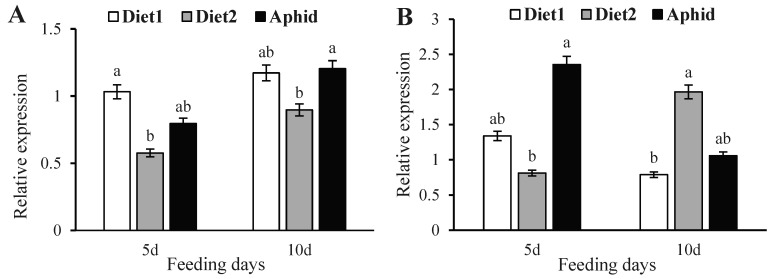
Relative expression levels of *Met* and *Kr-h1* in *Coccinella septempunctata* males at 5 and 10 d after feeding on diet 1, diet 2 (diet 1 + JH), or aphids. Relative expression of *Met* (**A**) and *Kr-h1* (**B**). Data points indicate means ± SDs, and columns labeled with different letters indicate significance at *p* < 0.05 using the LSD test.

*Met* expression was lower when male adults were fed with diet 2 compared to diet 1 and aphids (Figure 1A). *Met* expression levels in 5-day-old males fed with diet 2 were 44.17% and 27.55% lower than those fed with diet 1 and aphids, respectively (F = 11.2110, *p* = 0.0229). The expression levels of *Met* in 10-day-old male adults were 23.44% and 25.42% lower than those fed with diet 1 and aphids, respectively (F = 8.6224, *p* = 0.0354). In addition, *Met* expression levels in males increased after they were fed on different diets for 5–10 d.

*Kr-h1* expression in males fed on diet 2 for 5 d was 39.44% and 65.50% lower than those fed with diet 1 and aphids, respectively (F = 11.4387, *p* = 0.0221) (Figure 1B). *Kr-h1* expression in males increased 10 d after feeding on diet 2 and was 249.15% and 185.57% higher than those fed on diet 1 and aphids, respectively (F = 13.2867, *p* = 0.0171). Expression levels of *Kr-h1* in males increased from 5 to 10 d after feeding on diet 2 but decreased after feeding on diet 1 and aphids.

### 3.2. Effects of dsRNA Injection on Met and Kr-h1 Expression

**Figure 2:**
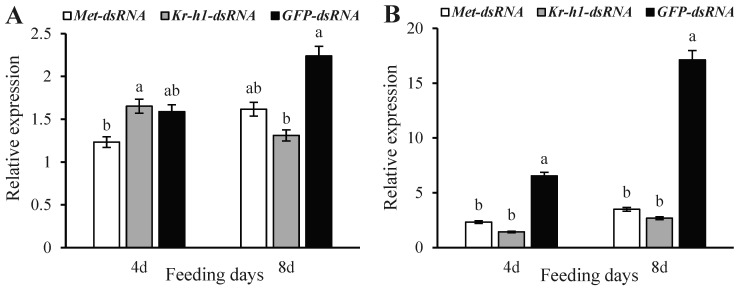
Relative expression levels of *Met* and *Kr-h1* in *Coccinella septempunctata* at 5 and 10 d after injection with Met-dsRNA, Kr-hl-dsRNA, and GFP-dsRNA. Relative expression of *Met* (**A**) and *Kr-h1* (**B**). Data represent means ± SDs, and columns labeled with different letters indicate significance at *p* < 0.05 using the LSD test.

Four days after injection with *Met*-dsRNA, *Met* expression was significantly reduced as compared to injection with *Kr-h1*-dsRNA and was 22.39% lower than the levels observed after injection with *GFP*-dsRNA (Figure 2A) (F = 1.0298, *p* = 0.0236). *Met* expression was increased by injection with *Kr-h1*-dsRNA, and there was no difference in the expression levels when compared to *GFP*-dsRNA-injected males (F = 0.5721, *p* = 0.5284). At 8 d after injection with *Met*-dsRNA and *Kr-h1*-dsRNA, *Met* expression decreased by 27.72% and 41.42%, respectively, when compared to the expression in *GFP*-dsRNA-injected males (F = 8.8996, *p* = 0.0337).

*Kr-h1* expression in males injected with *Met*-dsRNA and *Kr-h1*-dsRNA was significantly reduced after 4 d and was 64.41% and 78.13% lower than the levels in males injected with *GFP*-dsRNA, respectively (Figure 2B) (F = 7.4212, *p* = 0.0451). At 8 d after injection with *Met*-dsRNA and *Kr-h1*-dsRNA, *Kr-h1* expression in males remained significantly reduced and was 79.59% and 84.35% lower than the expression levels in *GFP*-dsRNA-injected males, respectively (F = 202.9907, *p* = 0.0001).

### 3.3. Effects of dsRNA Injection on Testes Development

**Figure 3:**
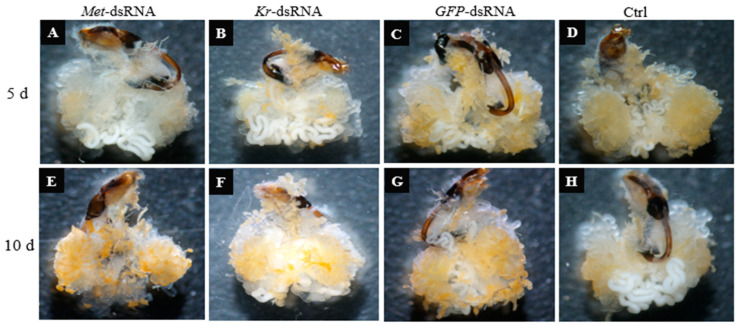
Testes development in *Coccinella septempunctata* after injection with *Met*-dsRNA, *Kr-h1*-dsRNA, and *GFP*-dsRNA. Panels (**A**–**D**) show testes in males at 5 d after microinjection with dsRNA. Panels (**E**–**H**) show testes in males at 10 d after dsRNA injection. The control panel (Ctrl) shows testes development in non-injected males.

Testes development in male ladybugs injected with *Met*-dsRNA and *Kr-h1*-dsRNA was obviously different at 5 and 10 d after injection as compared to those injected with *GFP*-dsRNA (Figure 3). The *GFP*-dsRNA-injected group had more white substances (it contains a large amount of semen proteins) in the accessory gland and vas deferens, while the groups injected with *Met*-dsRNA and *Kr-h1*-dsRNA had fewer white substances in the accessory gland and vas deferens and their testicular tubes were in a collapsed state.

**Figure 4:**
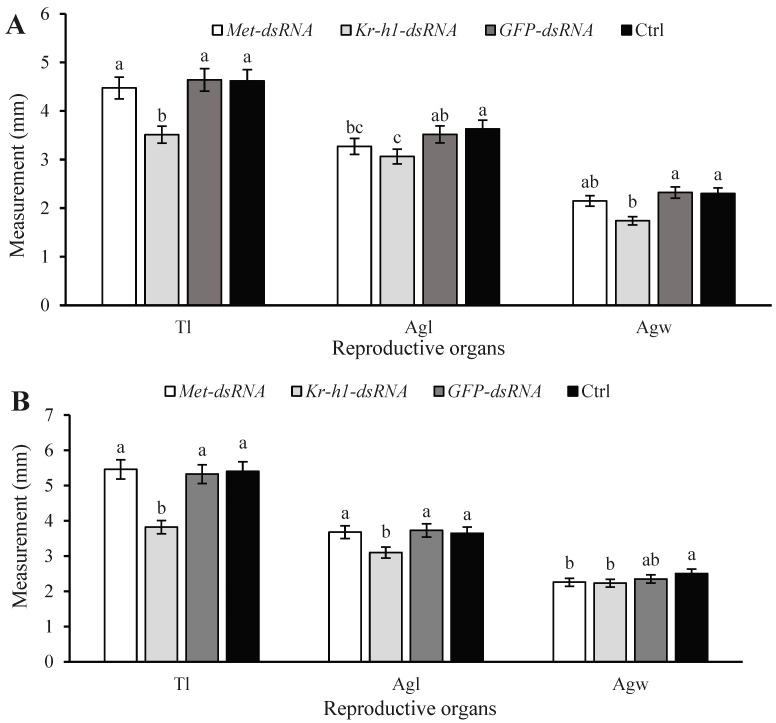
Testes measurements in *Coccinella septempunctata* after injection with *Met*-dsRNA, *Kr-h1*-dsRNA, and *GFP*-dsRNA. The Ctrl columns represent the non-injected control. (**A**) Testes measurements 5 d after microinjection and (**B**) testes measurements at 10 d. Abbreviations: Tl, testes length; Agl, accessory gland length; Agw, accessory gland width. Data represent means ± SDs. Columns labeled with different letters indicate significance at *p* < 0.05 using the LSD test.

When ladybugs were injected with *Met*-dsRNA and assessed at 5 d, testes lengths and accessory gland lengths and widths were 3.61%, 7.02%, and 7.53% lower, respectively, than the measurements in *GFP*-dsRNA-injected ladybugs (Figure 4A). When ladybugs were injected with *Kr-h1*-dsRNA and assessed at 5 d, testes lengths and accessory gland lengths and widths were 24.32%, 12.92%, and 25.08% lower, respectively, than those injected with *GFP*-dsRNA. When ladybugs were injected with *GFP*-dsRNA and assessed at 5 d, testes lengths and accessory gland lengths and widths were 0.46%, −3.04%, and 0.90% lower, respectively, than those of the non-injected groups (Figure 4A). There was no significant difference in testes lengths and accessory gland lengths and widths when comparing the *Met*-dsRNA- and the *GFP*-dsRNA-injected groups at 10 d (Figure 4B). However, when ladybugs were injected with *Kr-h1*-dsRNA and assessed at 10 d, the testes lengths and accessory gland lengths and widths were 28.30%, 16.85%, and 5.04% lower, respectively, than those injected with *GFP*-dsRNA (Figure 4B). When ladybugs were injected with *GFP*-dsRNA and assessed at 5 d, testes lengths and accessory gland lengths and widths were −1.39%, 2.26%, and −6.26% lower, respectively, than those of the non-injected groups (Figure 4B). There was no significant difference in testes lengths or accessory gland lengths and widths in the *GFP*-dsRNA-injected and non-injected groups, indicating that the dsRNA did not have an obvious effect.

### 3.4. Effects of dsRNA Injection on Egg Production

**Figure 5:**
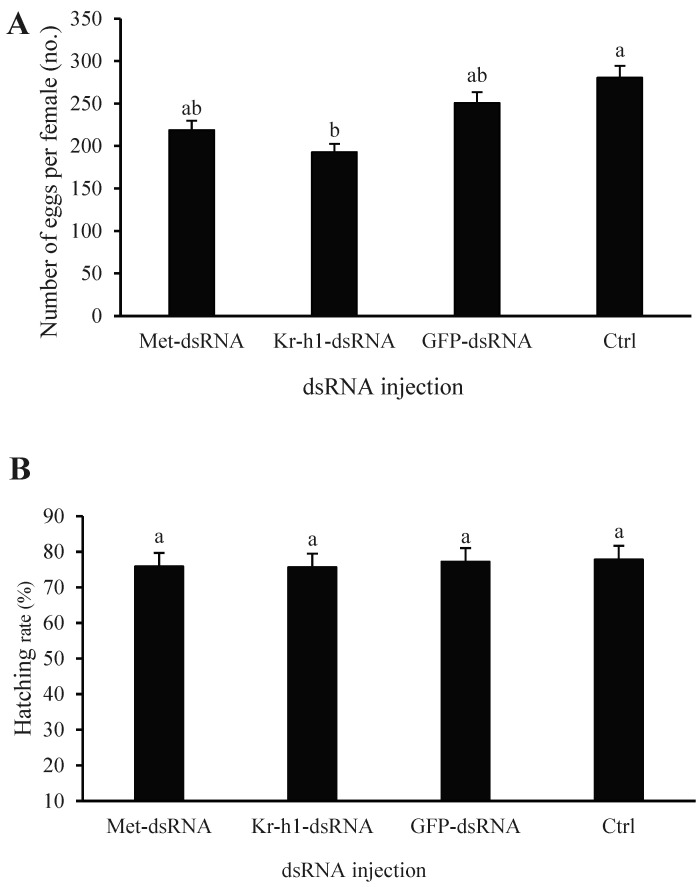
Fecundity of *Coccinella septempunctata* males after injection with *Met*-dsRNA, *Kr-h1*-dsRNA, and *GFP*-dsRNA and mating with female ladybugs. Ctrl, non-injected control. (**A**) Number of egg production; (**B**) Hatching rate. Data points represent means ± SDs. Columns labeled with different letters indicate significance at *p* < 0.05 using the LSD test.

Male ladybugs were injected with dsRNA, paired with females for mating, and egg production was monitored after 20 d. The microinjection of males with *Met*-dsRNA and *Kr-h1*-dsRNA resulted in an average of 219 and 193 eggs in paired females, respectively (Figure 5A), which was lower than the number of eggs produced when adult males were injected with *GFP*-dsRNA (*n* = 251) or were untreated (*n* = 281). In summary, the injection of male adults with *Met*-dsRNA and *Kr-h1*-dsRNA resulted in decreased egg production by females, whereas the injection of males with *GFP*-dsRNA did not have a significant effect on egg production in females (F = 4.8900, *p* = 0.0473). The hatching rates of male insects injected with *Met*-dsRNA and *Kr-h1*-dsRNA decreased when paired with females, but the difference was not significant compared to males injected with *GFP*-dsRNA and males that were not injected (F = 0.2491, *p* = 0.8593) (Figure 5B).

## 4. Discussion

The addition of JH to the artificial diet resulted in lower *Met* expression after 5 and 10 d of feeding, possibly due to the increased consumption of catabolism of the *Met* receptor in the JH metabolism pathway, resulting in decreased *Met* expression. A decrease in *Kr-h1* expression was also observed after 5 d of feeding; however, *Kr-h1* expression significantly increased after 10 d of feeding, indicating that *Kr-h1* expression can fluctuate in response to exogenous JH. When the JH titer is high, Met binds to JH, and the homodimer dissociates; then, Met enters the nucleus and induces the expression of the *Kr-h1* gene through the JH reaction element in the cooperation of heat shock protein Hsp83 and transport receptor Importin-β [28,29]. In Han et al.’s [14] studies, supplementation of *H. axyridis* females with JH promoted the upregulation of *Met* and *Kr-h1*, and injecting JH into newly emerged male adults of *Agrotis ipsilon* induced the expression of *Met* and *Kr-h1* [18].

JH was shown to promote the growth, development, and maturation of reproductive glands and enhance the production of glandular secretions in numerous male insects [15,16,28]. When *Tribolium castanenum* was supplied with exogenous JH, the volume of its accessory glands increased; however, excessive amounts of JH inhibited the normal physiological functions of the testes and accessory glands [16]. The injection of JH-III into young males of *Agrotis ipsilon* enhanced the behavioral responses to sex pheromones with increased *Met* and *Kr-h1* expression levels [30]. In contrast, the knockdown of *Met* led to decreased protein content in male accessory glands of *Pyrrhocoris apterus* [31]. Insufficient JH in *Drosophila melanogaster* led to decreased protein production in male accessory glands; furthermore, the deletion of *Met* weakened the physiological effects of JH and reduced protein accumulation in accessory glands [32]. In addition, exogenous hormones can indirectly regulate insect reproduction by influencing mating behavior, optic nerve differentiation, and hierarchical differentiation [33] and enhancing locomotory activity [34]. It is important to note that the regulation of optic nerve differentiation and mating behavior in adult *D. melanogaster* by JH is mainly achieved through *Met* [35]. In *Agrotis ipsilon*, *Kr-h1* may have a role in sexual maturation [36]. Knockdown of *Met* in *Spodoptera frugiperda* by RNAi decreased JHIII titers, delayed ovarian development, and reduced egg production [37]. In this study, the injection of *Met*-dsRNA and *Kr-h1*-dsRNA into male ladybugs reduced *Met* and *Kr-h1* expression, delayed testes development, reduced the volume of accessory glands, and decreased egg production in mated females. Although *Met*-dsRNA and *Kr-h1*-dsRNA injection downregulated the expression of *Met* and *Kr-h1* in male ladybugs, there was a slight decrease in male reproductive capacity relative to the dsGFP control, suggesting that dsGFP may have potential off-target effects. When *Kr-h1*-dsRNA was injected into male ladybugs, accessory gland volume and egg production were lower than those after the injection of *Met*-dsRNA. Our results show that *Kr-h1* had a greater impact on the regulation of reproduction in male ladybugs than *Met*, which warrants further study. Furthermore, our findings show that males exhibited normal mating behavior after injection with *Met*-dsRNA and *Kr-h1*-dsRNA; however, it remains unclear whether JH titers and male semen proteins are impacted.

## 5. Conclusions

In summary, the regulatory role of *Met* and *Kr-h1* on the reproductive capacity of *C. septempunctata* males was verified by RNAi and further illustrated the role of JH signaling pathways in the reproduction of insects. Furthermore, our results provide a theoretical basis for an improvement in *C. septempunctata* artificial diets by enriching the reproductive capacity of male ladybugs.

## Figures and Tables

**Table 1:** Primer sequences used in this study.

Primer Name	Primer Sequence	Annealing Temperature
Met-F Met-R	GGGTGAGAGTGATGAGCGTT GCAGCCAAATGTCGTTACCC	58.9
Kr-h1-F Kr-h1-R	AACCTTTCGAGTGCCCTGAAT ATGCCTCCTCCTGAACCTACT	58.3
Met-dsRNA-F Met-dsRNA-R	taatacgactcactatagggGATGAATCGACCGGAAAAGA taatacgactcactatagggAGCAAGGAGACGACGGTAGA	
Kr-h1-dsRNA-F Kr-h1-dsRNA-R	taatacgactcactatagggAAGGATCTCACCACCGACAC taatacgactcactatagggGGCTCCGTTTGTTCTGGTAA	
GFP-dsRNA-F GFP-dsRNA-R	taatacgactcactatagggGCCAACACTTGTCACTACTT taatacgactcactatagggGGAGTATTTTGTTGATAATGGTCTG	
Actin-F Actin-R	GATTCGCCATCCAGGACATCTC TCCTTGCTCAGCTTGTTGTAGTC	60.0

Lowercase letters represent the T7 promoter region.

## Data Availability

All data from this experiment are contained in this article.
